# Threshold Values of Sleep Spindles Features in Healthy Adults Using Scalp‐EEG and Associations With Sleep Parameters

**DOI:** 10.1002/acn3.70055

**Published:** 2025-04-21

**Authors:** Julien Coelho, Heloïse Degros, Jean‐Arthur Micoulaud‐Franchi, Patricia Sagaspe, Emmanuel d'Incau, Paul Galvez, Christian Berthomier, Pierre Philip, Jacques Taillard

**Affiliations:** ^1^ SANPSY, CNRS, UMR 6033, Hôpital Pellegrin Univ Bordeaux Bordeaux France; ^2^ Service Universitaire de Médecine du Sommeil CHU de Bordeaux Bordeaux France; ^3^ PHYSIP Paris France

**Keywords:** biomarker, electrophysiology, healthy adults, sleep spindle, threshold value

## Abstract

**Objective:**

Sleep spindles are an electrophysiological fingerprint of the sleeping human brain. They can be described in terms of duration, frequency, amplitude, and density, and vary widely according to age and sex. Spindles play a role in sleep and wake functions and are altered in several neurological and psychiatric disorders. This study established the first threshold values for sleep spindles in healthy adults using scalp‐EEG and explored their associations with other sleep parameters.

**Methods:**

This observational prospective study was conducted with 80 healthy participants stratified by age and sex (40.9 years, range 19–74, 50% females). All participants underwent in‐laboratory polysomnography. Sleep spindles during N2 were analyzed using an automated procedure and categorized as fast (> 13 Hz) or slow (≤ 13 Hz).

**Results:**

For fast spindles, the threshold values were duration (0.80–1.11 s), frequency (13.4–14.3 Hz), amplitude (5.2–15.2 μV), and density (1.0–5.8 spindles/min). For slow spindles, the values were duration (0.79–1.17 s), frequency (12.3–12.9 Hz), amplitude (4.1–13.2 μV), and density (0.03–3.15 spindles/min). From age 40 onwards, the density, amplitude, and duration of both types of spindles decreased; the amplitudes of both types of spindles were higher in females. Higher amplitude in fast spindles was associated with increased excessive daytime sleepiness and an increased proportion of slow‐wave sleep.

**Interpretation:**

This study provides the first threshold values for sleep spindle characteristics in healthy adults. The findings emphasize the importance of investigating spindles to develop innovative biomarkers for neurological and psychiatric disorders and to gain deeper insights into the functioning of the sleeping brain.

## 1 Introduction

Sleep spindles are distinct trains of sinusoidal wave bursts, characterized by a waxing and waning pattern, with a frequency between 11 and 16 Hz and a duration of 0.5–3 s in electroencephalograms (EEGs) of humans during sleep [1]. They significantly contribute to the sigma frequency band, which is commonly used to identify spindle dynamics, regulation, and properties [2]. They are most prominent during non‐rapid eye movement (NREM) sleep, particularly in stage 2 (N2), where they occur at a rate of 2–8 spindles/min [2]. They are less frequent in stage 3 (N3), with a rate of 1–6 spindles per min. They originate from the thalamocortical, corticothalamic, and reticular networks [3, 4, 5, 6], and exhibit individual characteristics including duration, frequency, amplitude, density, and other parameters [2]. They are characterized by high interindividual stability and variability [7], are influenced by genetic inheritance [2, 7, 8], and correlate with anatomical properties of the brain [9, 10]. Thus, they can be considered an electrophysiological fingerprint.

Sleep spindles play a crucial role in brain plasticity and in “offline” processes during sleep [11], such as learning, memory consolidation, and declarative learning. In particular, long‐term memory consolidation involves communication between the hippocampus, where memories are initially formed, and the neocortex, where they are stored for the long term [12, 13, 14, 15]. A key mechanism in the transmission of information between the hippocampus and neocortex is the coupling of EEG slow waves (cortex) with sleep spindles (thalamus) and high‐frequency hippocampal discharges (hippocampus) [14, 16, 17]. Alzheimer's disease is associated with changes in sleep patterns, particularly in the characteristics of sleep spindles, which become less frequent [18, 19, 20, 21] and occur at a lower frequency [21, 22]. Recently, we demonstrated that a decrease in the amplitude of sleep spindles is linked to a higher risk of developing moderate cognitive impairment in the elderly [23].

Thus, sleep spindles are an ideal candidate to serve as a noninvasive, easy‐to‐assess, and widely acceptable objective biomarker for evaluating sleep and wake functions, including memory consolidation, cognitive abilities, and various sleep, psychiatric, and neurological disorders [2]. In 2023, a cross‐sectional study conducted in a sleep clinic analyzed the sleep patterns of 567 children, providing the first threshold values for sleep spindle characteristics from birth to adulthood [24]. However, no previous study has established threshold values for sleep spindle features in healthy adults. These threshold measures could be instrumental in identifying pathology and monitoring pathological changes in disease populations and could significantly advance the discovery and diagnosis of neurodegenerative disorders.

In this context, several precautions are necessary. First, it is important to recognize that there are two types of sleep spindle—fast and slow—which appear to be unrelated and are typically distinguished by their frequency [25]. Fast spindles generally have a frequency between 13 and 16 Hz and are predominantly recorded over central and parietal regions, whereas slow spindles have a frequency of 11 to 13 Hz and have a frontal topography [26]. Each spindle type has distinct determinants and requires specific analysis [2]. Second, spectral power in sigma activity decreases from young adulthood to late adulthood [27, 28, 29] and is associated with sex differences [28]. Therefore, careful consideration is needed when constituting a balanced sample and adjusting the analyses. Third, sleep spindles are regulated by both circadian and homeostatic processes [2]. To minimize the impact on spindle characteristics, sleep–wake behaviors during the protocol must be standardized. In addition, understanding the association between spindle features and habitual sleep–wake behaviors could enhance our understanding of brain activity during sleep.

Based on previous findings, in this study, we establish threshold values for the features of fast and slow sleep spindles in healthy adults using scalp‐EEG. We also compared the characteristics of fast and slow sleep spindles, analyzed the trends in these features according to age and sex, and explored the associations between fast and slow sleep spindles and subjective sleep parameters and polysomnography (PSG) measures.

## 2 Methods

### 2.1 Participants

This study was part of an observational monocentric study that investigated associations between PSG measures, including sleep spindles, and interindividual differences in sustained attention (http://www.clinicaltrials.gov; registration number: NCT04596449). Eligible participants were individuals affiliated with a social security system, capable of understanding the study and providing written informed consent, available for the two required study visits, aged 18–75 years, and with a body mass index (BMI) between 18 and 27 kg/m^2^. Exclusion criteria included severe life‐threatening pathologies; uncontrolled endocrine disorders (e.g., dysthyroidism and diabetes); progressive cardiovascular conditions and related medications; progressive neurological conditions (treated or untreated) such as brain tumors, epilepsy, migraines, stroke, sclerosis, myoclonus, chorea, neuropathy, muscular dystrophies, and myotonic dystrophy; psychiatric disorders including depression, bipolar disorders, current anxiety, psychoses, and substance dependence; use of psychotropic medications; long‐term use of benzodiazepines and Z‐drugs (zolpidem and zopiclone); shift workers or night workers who have been on call or on call in the last 72 h; pregnant and/or breastfeeding patients; and individuals deprived of liberty by judicial or administrative decision or under curatorship or guardianship. Participants having subjective daytime sleepiness (Epworth scale score ≥ 11) or any suspicion of obstructive sleep apnea or other sleep disorders (i.e., any items from the Basic Nordic Sleep Questionnaire ≥ 4) were excluded to prevent physiological alterations from disrupting the behavioral and electrophysiological measurements. Recruitment was stratified by age and sex and took place from January 2021 to October 2021, a period that was outside the first wave of COVID‐19 in France but was marked by the closure of nurseries and schools in April 2021. Notably, none of the participants worked entirely remotely, and the level of sleep disturbances remained consistent during this period, according to *Santé Publique France* [30], a French public health organization. The protocol involved two visits over 3 weeks for each participant: an initial visit for questionnaire completion and an experimental visit that included various psychometric tasks and overnight PSG.

### 2.2 Measures

#### 2.2.1 Sociodemographic Characteristics

We collected basic sociodemographic data, including age (both as a continuous variable and categorized as < 20, 20–40, and > 40 years), sex (female and male), and BMI. Being overweight was defined as BMI ≥ 25 kg/m^2^.

#### 2.2.2 Subjective Sleep Parameters

The Morningness–Eveningness Questionnaire (MEQ) was used to assess participants' chronotype preference [31]. This questionnaire consists of 19 items rated on a four‐ or five‐point Likert scale. Aggregate scores range from 16 to 86, with higher scores indicating a stronger morning chronotype. The French version of the MEQ, which has been validated for internal and external consistency [32], categorizes individuals under 45 years old as evening chronotype (scores < 42), neutral (scores between 42 and 57), or morning chronotype (scores > 57). For individuals aged 45 years and older, modified thresholds are applied: evening chronotype < 53 and morning chronotype > 64 [32].

Participants also completed the French version of the Munich Chronotype Questionnaire, reporting their typical sleep behaviors over the prior 4 weeks, including bedtimes and rise times on workdays and free days [33]. These data were used to calculate social jetlag. Corrections were applied to account for sleep debt, and social jetlag was considered significant if there was at least a 1 h shift [34, 35, 36].

The Epworth Sleepiness Scale (ESS) was used to assess participants' level of excessive daytime sleepiness (EDS) [37]. The scale includes eight situations, each rated from 0 (no chance of dozing) to 3 (high chance of dozing). The total score ranges from 0 to 24, with higher scores indicating more severe EDS. The French version of the ESS, which has demonstrated good psychometric properties, was utilized [38]. Scores ≥ 11 were considered indicative of moderate EDS.

#### 2.2.3 Polysomnography Measures

Participants underwent level one, overnight, technologist‐monitored, in‐laboratory PSG using a Brainbox EEG‐1042 digital sleep recorder (Braintronics, Almere, The Netherlands; resolution 16‐bit, stop band frequency 100 Hz, passband ripple 0.027 dB, stopband ripple −40 dB). The PSG was conducted according to American Academy of Sleep Medicine (AASM) guidelines [1] and followed standard recording and scoring procedures. Five Ag‐AgCl electrodes were placed according to the international 10–20 system (F3, C3, O1, Cz, Pz) and referenced to linked mastoids. In addition, an electrooculogram, two chin electromyograms, and an electrocardiogram were recorded. Participants were allowed to choose their bedtime freely, but rise times on weekdays were constrained by professional commitments. On the day of the PSG, participants were instructed to maintain their usual routines regarding food, caffeine, smoking, and alcohol consumption.

Sleep staging (W, N1, N2, N3, R) and event scoring were conducted by an experienced polysomnographic technologist following AASM criteria. The sleep architecture parameters assessed included total sleep time, sleep latency, wake after sleep onset, frequency of micro‐awakenings per hour, sleep efficiency (percentage of total time in bed spent asleep), the proportions of each sleep stage (% N1, % N2, % N3, % R), and REM sleep latency.

#### 2.2.4 Sleep Spindle Detection and Feature Analysis

Sleep spindles were automatically detected using a previously published method (Aseega software, Physip, Paris, France) [39, 40, 41] during visually labeled N2 epochs from C3‐A2 and F3‐A2 derivations. The scalp‐EEG signal during N2 was bandpass filtered between 10.5 and 17.5 Hz using a linear phase FIR filter (−3 dB at 10 and 16 Hz) to adapt to all variations due to aging in particular. All sigma burst events lasting between 0.5 and 3 s were identified as spindles. Spindle detection did not rely on the absolute amplitude of the EEG signal, as it varies based on factors such as age, electrode placement, and skull morphology [42, 43, 44]. Instead, it was based on envelope activity after computing the mean level in the sigma sub‐band. The pairwise agreement between ASEEGA scoring (from the Cz‐Pz derivation) and visual scoring was 80%, with Cohen's kappa = 0.72, confirming optimal spindle detection.

Automatic detection of sleep spindles enables the measurement of several spindle characteristics, including duration (time from the beginning to the end of the event, expressed in s), frequency (mean instantaneous frequency during the event, expressed in Hz), amplitude (maximum amplitude of the sigma‐filtered signal during the event, expressed in μV), density (number of spindles per min during N2), frequential instability (the variance of instantaneous frequency during the event, estimated by the time derivative of the analytic signal's phase associated with the sigma output, following the method of Nir et al. [45], expressed in Hz^2^, with higher values indicating greater frequential instability), and frequential purity (the spindle's visibility in the EEG trace for an expert, estimated as the normalized sigma power of the event P_sigma_/P_eeg_, expressed as a percentage, with higher values indicating easier visual detection within the EEG frequency mix). The characteristics of slow spindles (frequency ≤ 13 Hz) were calculated from the F3‐A2 derivation and were therefore considered frontal slow spindles, whereas the characteristics of fast spindles (frequency > 13 Hz) were calculated from the C3‐A2 derivation and were thus considered central fast spindles.

#### 2.2.5 Statistical Analysis

Descriptive statistics for the collected data included frequencies (n) and percentages (%) for categorical variables, and medians (m) with interquartile ranges (Q1–Q3) for continuous variables. The Wilcoxon‐Mann–Whitney test was used to compare features between fast and slow sleep spindles. Our detection method (i.e., instantaneous frequency measurement) minimizes frequency resolution issues, thereby ensuring the validity of statistical comparisons. For the primary objective, the 5th, 10th, 25th, 75th, 90th, and 95th percentiles were calculated to describe threshold values for each sleep spindle characteristic. For the first secondary objective, univariate associations between age and sleep spindle features were analyzed using linear regression with a likelihood ratio test, while univariate associations between sex and sleep spindle features were examined using the Wilcoxon‐Mann–Whitney test. For the second secondary objective, multivariate linear regression models, adjusted for age and sex, were used to analyze the relationships among features between fast and slow sleep spindles (dependent variables) and subjective sleep and PSG measures (explanatory variables). Data were analyzed using R version 4.1.2 (GUI 1.77 High Sierra build 8007), with a significance level set at 5%.

## 3 Results

### 3.1 Sample Description

**FIGURE 1:**
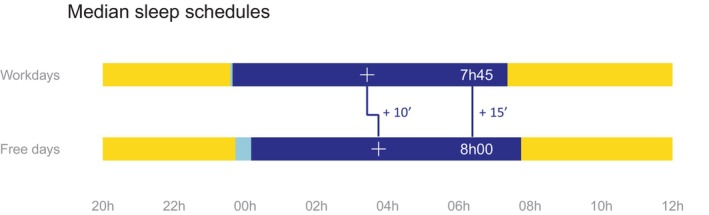
Median sleep schedules. Median sleep schedules of 80 participants. Yellow: Awake, Light blue: Sleep latency, Dark blue: Sleep. White cross: Mid‐sleep point and relative difference during workdays and free days. HHhHH: Total sleep time and relative difference during workdays and free days.

The study included 80 participants (mean age: 40.9 years, range 19–74; 50% female; 22.5% overweight). Based on sleep diaries, the median bedtimes and rise times were 11:35 p.m. and 7:22 a.m. on workdays, and 11:44 p.m. and 7:45 a.m. on free days, respectively (Figure 1). Eight participants (14.5%) reported short sleep durations (< 7 h) and four (5.0%) exhibited EDS. According to the MEQ, 11 participants (14.1%) were classified as morning types and 10 (12.8%) were evening types. Additional descriptive information, including PSG measures, is provided in Table 1.

**TABLE 1:** Sample characteristics.

Variables	*n* (%)	Median [IQ]
*Sociodemographic characteristics*		
Age		40.9 [23.1–53.0]
18–40 years	39 (48.8%)	
40–60 years	27 (33.8%)	
60 years or more	14 (17.5%)	
Sex: Female	40 (50.0%)	
BMI		22.8 [21.5–24.7]
Overweight (BMI ≥ 25)	18 (22.5%)	
*Subjective sleep parameters*		
Chronotype (MEQ)		55 [49–58]
Morning chronotype	11 (14.1%)	
Neutral chronotype	57 (73.1%)	
Evening chronotype	10 (12.8%)	
Corrected social jetlag (MCTQ)		45 min [17.5 min—60 min]
Significant (≥ 60 min)	11 (17.5%)	
Excessive daytime sleepiness (ESS)		6.5 [4–8]
Moderate (ESS ≥ 11)	4 (5.0%)	
*Polysomnography measures*		
Total sleep time		445 [405–474]
Sleep latency		12 [5.5–19]
WASO		32 [21.5–68.5]
Micro‐awakenings		6.6 [4.7–9.6]
Sleep efficiency		90.7% [82.2%–93.3%]
% N1		4.0% [2.7%–5.8%]
% N2		52.1% [47.1%–56.6%]
% N3		20.6% [17.2%–24.6%]
% R		22.0% [18.2%–24.4%]
REM sleep latency		83 [65.5–126.5]

### 3.2 Sleep Spindle Features

**FIGURE 2:**
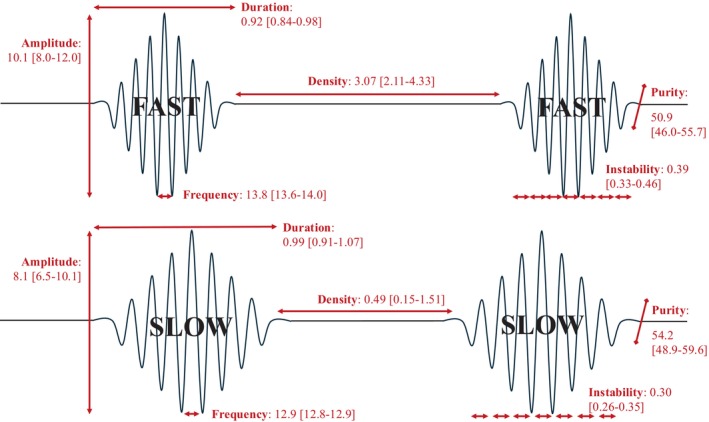
Median spindles features. Median spindle features of 80 participants. Upper panel: Fast sleep spindles (frequency > 13 Hz). Lower part: Slow sleep spindles (frequency ≤ 13 Hz). Duration: Time between the beginning and the end of the sleep spindles (s). Frequency: Number of oscillations per s (Hertz). Amplitude: Average size of oscillations (μV). Density: Number of spindles during N2 (per min). Frequency instability: Average fluctuations around the spindle frequency. Frequency purity: Degree of spindle “visibility” on EEG oscillations.

Figure 2 presents the features of the sleep spindles.

**FIGURE 3:**
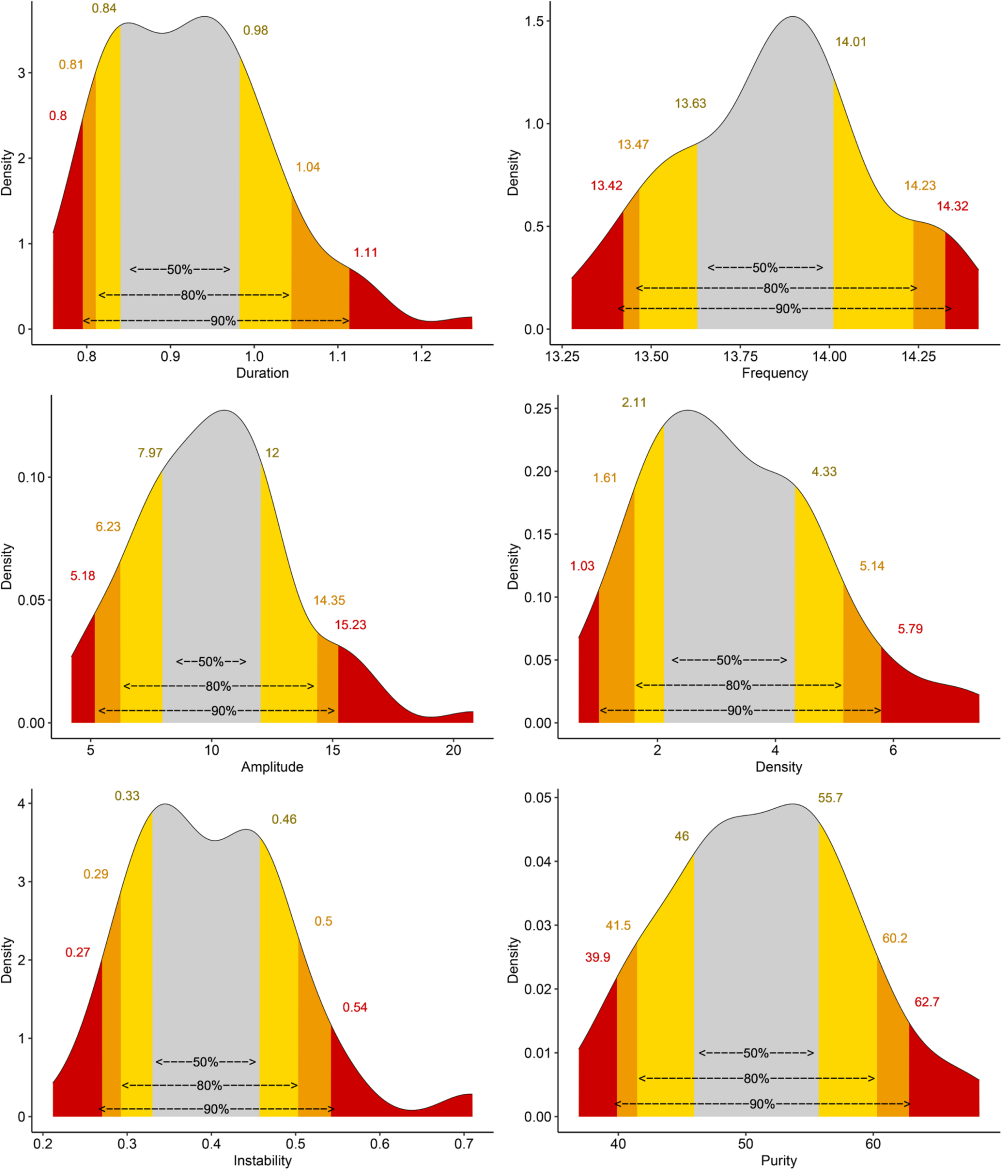
Threshold values of central fast sleep spindle features. Threshold values of central fast sleep spindle features. Gray: Values between the 25th and 75th percentiles. Yellow: Values between the 10th and 25th percentiles or between the 75th and 90th percentiles. Orange: Values between the 5th and 10th percentiles or between the 90th and 95th percentiles. Red: Values below the 5th percentile or above the 95th percentile.

**FIGURE 4:**
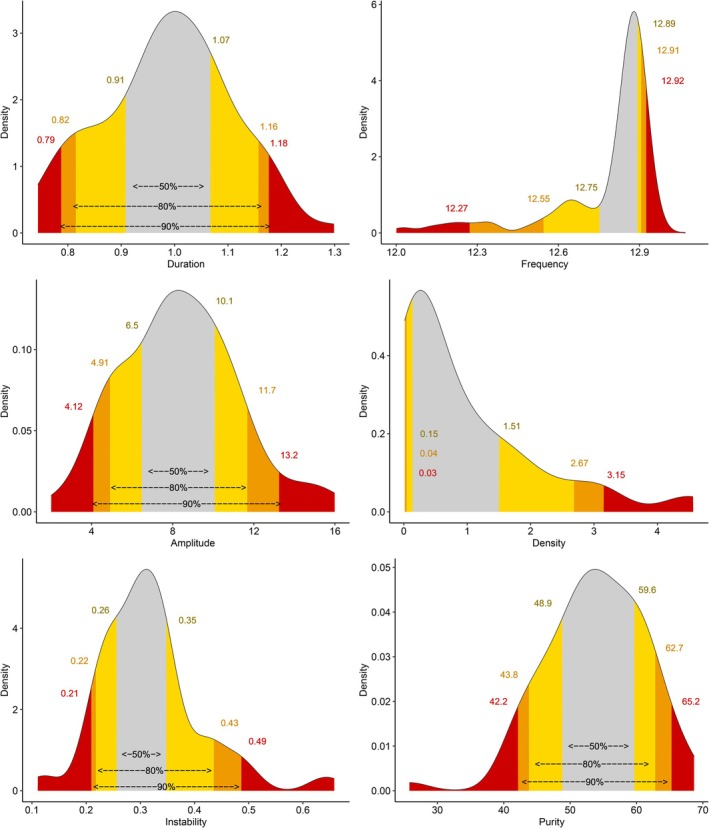
Threshold values of slow sleep spindle features. Threshold values of frontal slow sleep spindle features. Gray: Values between the 25th and 75th percentiles. Yellow: Values between the 10th and 25th percentiles or between the 75th and 90th percentiles. Orange: Values between the 5th and 10th percentiles or between the 90th and 95th percentiles. Red: Values below the 5th percentile or above the 95th percentile.

**FIGURE 5:**
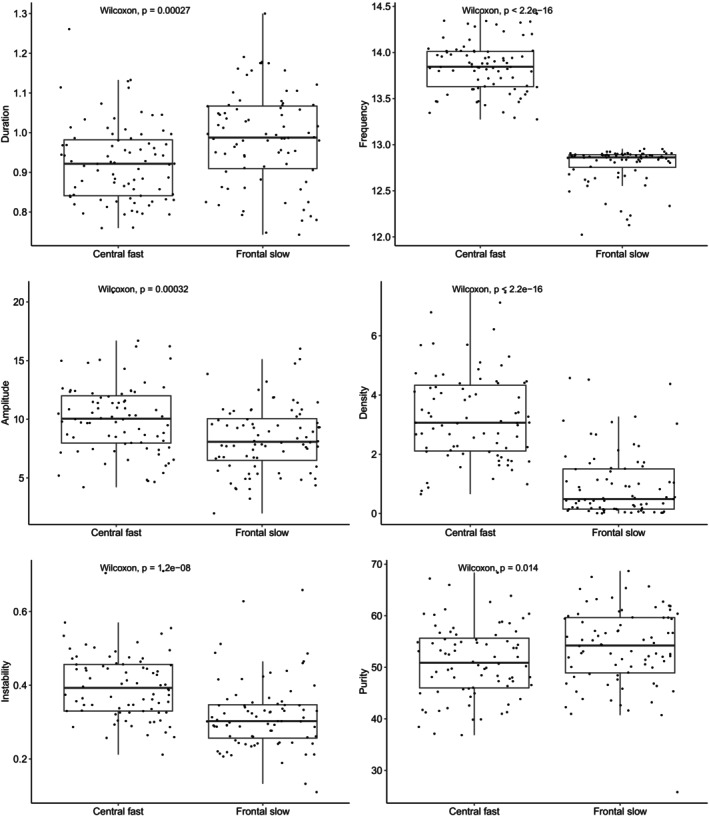
Differences in features between fast and slow sleep spindles. Boxplot of differences in features between fast and slow sleep spindles. Sleep spindle features in each subgroup were compared using the Wilcoxon‐Mann‐Whitney test. The level of significance was set at 0.05.

For central fast sleep spindles, 90% of the duration values ranged between 0.80 and 1.11 s, 90% of the frequency values ranged between 13.4 and 14.3 Hz, 90% of the amplitude values ranged between 5.2 and 15.2 μV, 90% of the density values ranged between 1.0 and 5.8 per min of N2, 90% of the frequential instability values ranged between 0.27 and 0.54, and 90% of the frequential purity values ranged between 39.9% and 62.7% (Figure 3). The values for frontal slow spindles were 0.79–1.18 s, 12.3–12.9 Hz, 4.1–13.2 μV, 0.03%–3.15%, 0.21%–0.49%, and 42.2%–65.2%, respectively (Figure 4). Thus, central fast spindles were shorter (0.92 vs. 0.99 s, *p* < 0.001), denser (3.07 vs. 0.49 per minutes of N2, *p* < 0.001), and had higher amplitudes (10.1 vs. 8.1 μV, *p* < 0.001) and frequential instability (0.39 vs. 0.30, p < 0.001) but lower frequential purity (50.9 vs. 54.2, *p* = 0.014) than slow ones (Figure 5).

### 3.3 Associations Among Age, Sex, and Sleep Spindle Features

**FIGURE 6:**
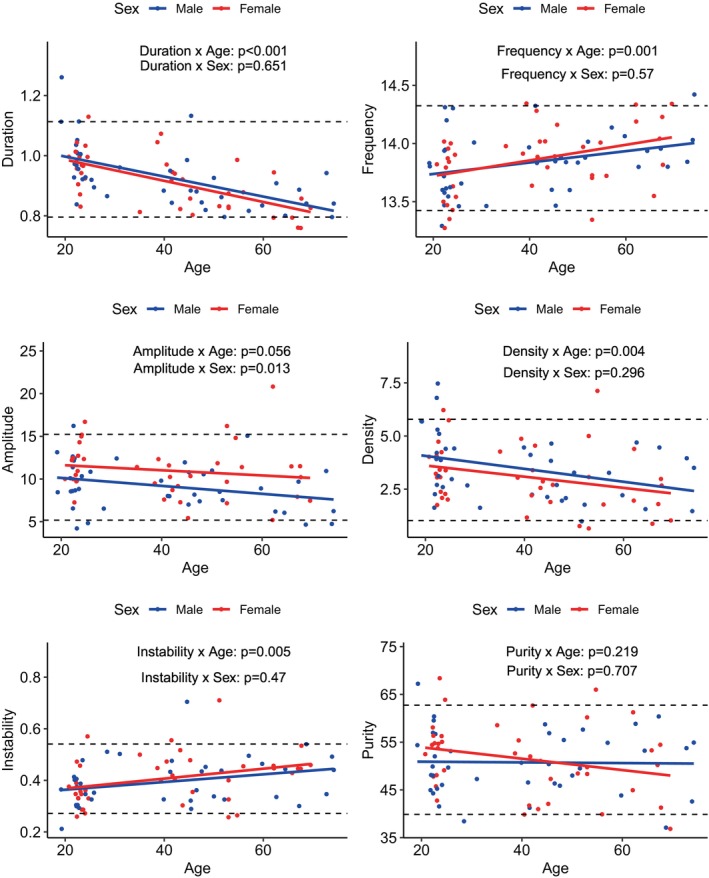
Associations among age, sex, and central fast sleep spindle features. Dot plot of the central fast sleep spindle features according to the age and sex of participants. Dashed lines: 90% threshold value. Sleep spindle features were compared using linear regression with likelihood ratio test for age and a Wilcoxon‐Mann–Whitney test for sex. The level of significance was set at 0.05.

**FIGURE 7:**
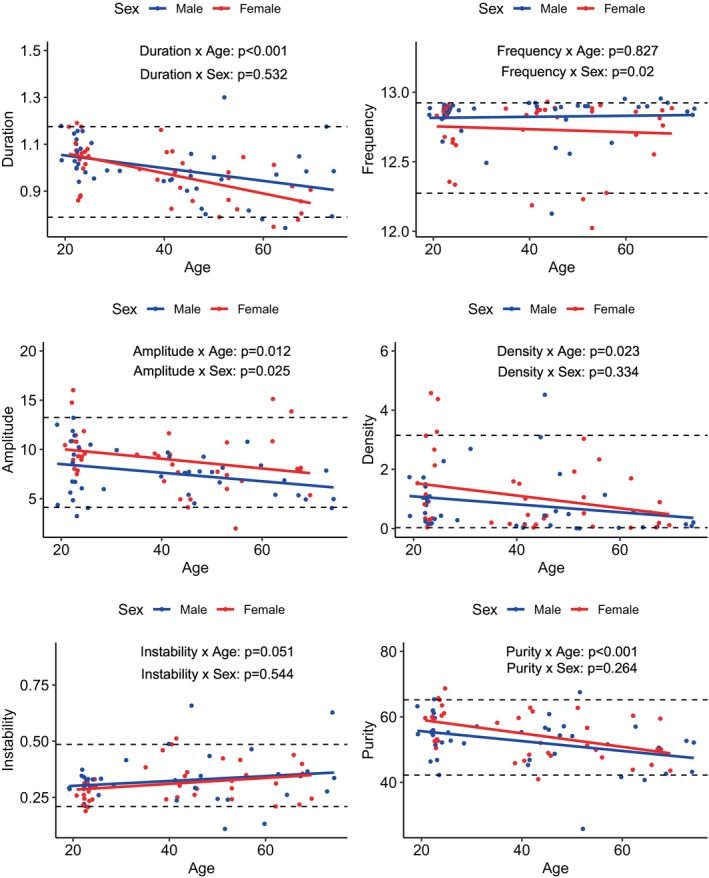
Associations among age, sex, and frontal slow sleep spindle features. Dot plot of the frontal slow sleep spindle features according to the age and sex of participants. Dashed lines: 90% threshold value. Sleep spindle features were compared using linear regression with likelihood ratio test for age and a Wilcoxon‐Mann–Whitney test for sex. The level of significance was set at 0.05.

Results are presented in Figures 6 and 7. For central fast spindles, the frequency (*p* = 0.001) and frequential instability (*p* = 0.005) increased with age while the duration (*p* < 0.001) and density (*p* = 0.004) decreased, and the amplitude tended to decrease (*p* = 0.056). Regarding sex, amplitude was higher in females (*p* = 0.013). For frontal slow spindles, the duration (*p* < 0.001), amplitude (*p* = 0.012), density (*p* = 0.023), and frequential purity (*p* < 0.001) decreased with age. Frequency was higher in males (*p* = 0.020) and amplitude was higher in females (*p* = 0.025). Overall, associations were linear, with spindle features changing notably from age 40 onwards.

### 3.4 Associations Among Subjective Sleep Parameters, Polysomnography Measures, and Sleep Spindle Features

Results are presented in Tables 2 and 3. All analyses were adjusted for age and sex. Regarding subjective sleep measures, morningness was associated with a longer duration of central fast sleep spindles (*ß* = 0.33, *p* = 0.025), higher density of frontal slow sleep spindles (*ß* = 0.38, *p* = 0.034), and greater frequential purity for both spindle types (fast: *ß* = 0.42, *p* = 0.018 and slow: *ß* = 0.38, *p* = 0.022). EDS was associated with a higher amplitude of central fast spindles (*ß* = 0.08, *p* = 0.038). Regarding PSG measures, greater frequential instability was associated with longer total sleep time for frontal slow spindles (*ß* = 0.38, *p* = 0.021) and longer sleep latency for fast ones (*ß* = 0.17, *p* = 0.032). A high frequency of micro‐awakenings was associated with a higher frequency of frontal slow spindles (*ß* = 0.07, *p* = 0.016). A higher proportion of N1 sleep was associated with a longer duration of central fast spindles (*ß* = 0.72, *p* = 0.024). A higher proportion of N2 was associated with a lower amplitude (*ß* = −0.46, *p* = 0.003) and lower density (*ß* = −0.37, *p* = 0.021) of frontal slow spindles. A higher proportion of N3 was associated with a higher amplitude of both types of spindle (fast: *ß* = 0.50, *p* = 0.009 and slow: *ß* = 0.65, *p* = 0.001), as well as a lower frequency (*ß* = −0.43, *p* = 0.036) and greater density (*ß* = 0.45, *p* = 0.024) of frontal slow spindles. Longer REM sleep latency was associated with greater frequential instability (*ß* = 0.05, *p* = 0.028) and lower frequential purity (*ß* = −0.05, *p* = 0.016) of central fast sleep spindles. No spindle features varied with social jetlag or the proportion of REM sleep.

**TABLE 2:** Adjusted associations among subjective sleep parameters, polysomnography measures, and central fast sleep spindle features.

	Duration	Frequency	Amplitude	Density	Instability	Purity
*Subjective sleep measures*						
Chronotype (MEQ)^a^	***ß* = 0.33** **[0.04; 0.61]** ***p* = 0.025**	*ß* = −0.06 [−0.4; 0.29] *p* = 0.733	*ß* = 0.15 [−0.19; 0.49] *p* = 0.375	*ß* = 0.28 [−0.06; 0.63] *p* = 0.102	*ß* = 0.05 [−0.3; 0.4] *p* = 0.779	***ß* = 0.42** **[0.08; 0.77]** ***p* = 0.018**
Social jetlag (MCTQ)^a^	*ß* = −0.03 [−0.09; 0.04] *p* = 0.435	*ß* = 0 [−0.08; 0.08] *p* = 0.968	*ß* = −0.03 [−0.11; 0.04] *p* = 0.396	*ß* = −0.03 [−0.11; 0.05] *p* = 0.471	*ß* = 0.02 [−0.04; 0.09] *p* = 0.463	*ß* = −0.06 [−0.14; 0.02] *p* = 0.137
Excessive daytime sleepiness (ESS)	*ß* = 0.02 [−0.04; 0.09] *p* = 0.46	*ß* = −0.01 [−0.08; 0.07] *p* = 0.823	***ß* = 0.08** **[0; 0.15]** ***p* = 0.038**	*ß* = 0.04 [−0.04; 0.11] *p* = 0.313	*ß* = −0.05 [−0.13; 0.02] *p* = 0.169	*ß* = 0.07 [0.00; 0.15] *p* = 0.061
*Polysomnography measures*						
Total sleep time (in hour)	*ß* = −0.08 [−0.35; 0.19] *p* = 0.554	*ß* = 0.01 [−0.3; 0.33] *p* = 0.932	*ß* = 0.21 [−0.1; 0.52] *p* = 0.188	*ß* = 0.27 [−0.05; 0.58] *p* = 0.096	*ß* = 0.08 [−0.25; 0.4] *p* = 0.643	*ß* = 0.18 [−0.15; 0.51] *p* = 0.284
Sleep latency^a^	*ß* = 0.01 [−0.13; 0.14] *p* = 0.932	*ß* = 0.09 [−0.07; 0.25] *p* = 0.29	*ß* = −0.06 [−0.22; 0.1] *p* = 0.46	*ß* = −0.07 [−0.24; 0.09] *p* = 0.373	***ß* = 0.17** **[0.02; 0.33]** ***p* = 0.032**	*ß* = −0.09 [−0.26; 0.08] *p* = 0.301
WASO^a^	*ß* = 0.01 [−0.04; 0.07] *p* = 0.593	*ß* = −0.02 [−0.08; 0.05] *p* = 0.577	*ß* = −0.02 [−0.09; 0.04] *p* = 0.467	*ß* = −0.06 [−0.12; 0.00] *p* = 0.068	*ß* = 0.05 [−0.02; 0.11] *p* = 0.148	*ß* = −0.02 [−0.09; 0.05] *p* = 0.541
Micro‐awakenings^a^	*ß* = 0 [−0.05; 0.05] *p* = 0.968	*ß* = −0.01 [−0.06; 0.05] *p* = 0.801	*ß* = −0.05 [−0.1; 0] *p* = 0.075	*ß* = −0.02 [−0.07; 0.04] *p* = 0.485	*ß* = −0.05 [−0.11; 0] *p* = 0.058	*ß* = −0.02 [−0.08; 0.03] *p* = 0.42
Sleep efficiency^a^	*ß* = −0.07 [−0.32; 0.18] *p* = 0.562	*ß* = 0.08 [−0.21; 0.37] *p* = 0.593	*ß* = 0.13 [−0.16; 0.42] *p* = 0.38	*ß* = 0.27 [−0.02; 0.56] *p* = 0.063	*ß* = −0.24 [−0.53; 0.05] *p* = 0.098	*ß* = 0.07 [−0.23; 0.38] *p* = 0.628
% N1^a^	***ß* = 0.72** **[0.1; 1.34]** ***p* = 0.024**	*ß* = 0.12 [−0.63; 0.87] *p* = 0.75	*ß* = −0.35 [−1.09; 0.39] *p* = 0.344	*ß* = 0.17 [−0.58; 0.92] *p* = 0.648	*ß* = −0.04 [−0.79; 0.72] *p* = 0.925	*ß* = 0.11 [−0.68; 0.89] *p* = 0.788
% N2^a^	*ß* = −0.21 [−0.48; 0.05] *p* = 0.11	*ß* = 0.07 [−0.24; 0.39] *p* = 0.645	*ß* = −0.39 [−0.69;−0.09] *p* = 0.011	*ß* = 0.03 [−0.29; 0.34] *p* = 0.860	*ß* = −0.16 [−0.47; 0.16] *p* = 0.327	*ß* = −0.09 [−0.41; 0.24] *p* = 0.601
% N3^a^	*ß* = 0.32 [−0.01; 0.65] *p* = 0.056	*ß* = −0.22 [−0.61; 0.17] *p* = 0.264	***ß* = 0.5** **[0.13; 0.87]** ***p* = 0.009**	*ß* = −0.2 [−0.6; 0.19] *p* = 0.300	*ß* = 0.28 [−0.11; 0.67] *p* = 0.161	*ß* = −0.07 [−0.48; 0.34] *p* = 0.732
% R^a^	*ß* = −0.19 [−0.53; 0.15] *p* = 0.267	*ß* = 0.07 [−0.33; 0.47] *p* = 0.727	*ß* = 0.22 [−0.17; 0.62] *p* = 0.263	*ß* = 0.11 [−0.29; 0.51] *p* = 0.590	*ß* = −0.02 [−0.43; 0.38] *p* = 0.903	*ß* = 0.17 [−0.25; 0.58] *p* = 0.421
REM sleep latency^a^	*ß* = 0.01 [−0.03; 0.04] *p* = 0.763	*ß* = 0.02 [−0.03; 0.06] *p* = 0.398	*ß* = −0.01 [−0.06; 0.03] *p* = 0.553	*ß* = −0.03 [−0.08; 0.01] *p* = 0.142	***ß* = 0.05** **[0.01; 0.09]** ***p* = 0.028**	***ß* =** −**0.05** **[−0.1; −0.01]** ***p* = 0.016**

*Note:* Multivariate linear regression models adjusted for age and sex with standardized value of sleep spindles features (mean = 0 and standard‐deviation = 1). *ß* [95% CI] *p*‐value. Significant associations in bold.

^a^ For 10 unit.

**TABLE 3:** Adjusted associations among subjective sleep parameters, polysomnography measures, and frontal slow sleep spindle features.

	Duration	Frequency	Amplitude	Density	Instability	Purity
*Subjective sleep measures*						
Chronotype (MEQ)^a^	*ß* = −0.05 [−0.37; 0.27] *p* = 0.76	*ß* = −0.3 [−0.63; 0.04] *p* = 0.083	*ß* = −0.05 [−0.39; 0.29] *p* = 0.763	***ß* = 0.38** **[0.03; 0.73]** ***p* = 0.034**	*ß* = 0 [−0.35; 0.35] *p* = 0.998	***ß* = 0.38** **[0.06; 0.7]** ***p* = 0.022**
Social jetlag (MCTQ)^a^	*ß* = −0.02 [−0.1; 0.05] *p* = 0.53	*ß* = −0.01 [−0.08; 0.06] *p* = 0.782	*ß* = −0.04 [−0.12; 0.04] *p* = 0.309	*ß* = −0.04 [−0.11; 0.04] *p* = 0.375	*ß* = 0.03 [−0.05; 0.11] *p* = 0.417	*ß* = −0.06 [−0.14; 0.01] *p* = 0.094
Excessive Daytime Sleepiness (ESS)	*ß* = 0 [−0.07; 0.07] *p* = 0.94	*ß* = 0.02 [−0.06; 0.1] *p* = 0.559	*ß* = −0.01 [−0.08; 0.07] *p* = 0.846	*ß* = 0 [−0.08; 0.08] *p* = 0.972	*ß* = −0.02 [−0.1; 0.06] *p* = 0.636	*ß* = 0.05 [−0.03; 0.12] *p* = 0.208
*Polysomnography measures*						
Total Sleep Time (in hour)	*ß* = 0 [−0.29; 0.3] *p* = 0.975	*ß* = −0.23 [−0.56; 0.1] *p* = 0.175	*ß* = −0.25 [−0.56; 0.07] *p* = 0.122	*ß* = 0.18 [−0.14; 0.51] *p* = 0.263	***ß* = 0.38** **[0.06; 0.7]** ***p* = 0.021**	*ß* = 0.17 [−0.13; 0.48] *p* = 0.26
Sleep latency^a^	*ß* = −0.12 [−0.27; 0.03] *p* = 0.105	*ß* = 0.05 [−0.12; 0.22] *p* = 0.592	*ß* = 0.05 [−0.12; 0.21] *p* = 0.569	*ß* = −0.09 [−0.25; 0.08] *p* = 0.284	*ß* = 0.09 [−0.08; 0.26] *p* = 0.286	*ß* = −0.11 [−0.27; 0.04] *p* = 0.16
WASO^a^	*ß* = −0.01 [−0.07; 0.05] *p* = 0.767	*ß* = 0.01 [−0.06; 0.08] *p* = 0.764	*ß* = 0.05 [−0.01; 0.12] *p* = 0.09	*ß* = −0.02 [−0.09; 0.04] *p* = 0.527	*ß* = 0 [−0.07; 0.07] *p* = 0.954	*ß* = −0.01 [−0.08; 0.05] *p* = 0.666
Micro‐awakenings^a^	*ß* = 0.02 [−0.03; 0.07] *p* = 0.431	***ß* = 0.07** **[0.01; 0.12]** ***p* = 0.016**	*ß* = −0.02 [−0.07; 0.04] *p* = 0.58	*ß* = −0.04 [−0.1; 0.01] *p* = 0.130	*ß* = −0.03 [−0.09; 0.03] *p* = 0.274	*ß* = 0.01 [−0.05; 0.06] *p* = 0.836
Sleep efficiency^a^	*ß* = 0.07 [−0.2; 0.34] *p* = 0.631	*ß* = −0.03 [−0.34; 0.28] *p* = 0.842	*ß* = −0.22 [−0.51; 0.06] *p* = 0.123	*ß* = 0.09 [−0.21; 0.39] *p* = 0.546	*ß* = 0.06 [−0.25; 0.36] *p* = 0.701	*ß* = 0.08 [−0.2; 0.36] *p* = 0.559
% N1^a^	*ß* = 0.05 [−0.64; 0.75] *p* = 0.88	*ß* = 0.3 [−0.48; 1.08] *p* = 0.448	*ß* = 0.01 [−0.74; 0.75] *p* = 0.987	*ß* = 0.1 [−0.67; 0.86] *p* = 0.805	*ß* = 0.19 [−0.6; 0.97] *p* = 0.636	*ß* = 0.18 [−0.54; 0.91] *p* = 0.614
% N2^a^	*ß* = −0.01 [−0.3; 0.28] *p* = 0.923	*ß* = 0.3 [−0.02; 0.62] *p* = 0.064	***ß* =** −**0.46** **[−0.75; −0.17]** ***p* = 0.003**	***ß* =** −**0.37** **[−0.68; −0.06]** ***p* = 0.021**	*ß* = −0.19 [−0.52; 0.14] *p* = 0.249	*ß* = −0.13 [−0.43; 0.18] *p* = 0.408
% N3^a^	*ß* = 0.17 [−0.19; 0.54] *p* = 0.339	***ß* =** −**0.43** **[−0.83; −0.03]** ***p* = 0.036**	***ß* = 0.65** **[0.29; 1.01]** ***p* = 0.001**	***ß* = 0.45** **[0.06; 0.84]** ***p* = 0.024**	*ß* = 0.33 [−0.08; 0.73] *p* = 0.109	*ß* = −0.01 [−0.39; 0.37] *p* = 0.94
% R^a^	*ß* = −0.17 [−0.54; 0.19] *p* = 0.353	*ß* = −0.14 [−0.55; 0.28] *p* = 0.511	*ß* = 0.07 [−0.33; 0.47] *p* = 0.72	*ß* = 0.11 [−0.3; 0.51] *p* = 0.606	*ß* = −0.09 [−0.5; 0.33] *p* = 0.686	*ß* = 0.16 [−0.23; 0.54] *p* = 0.414
REM sleep latency^a^	*ß* = −0.03 [−0.07; 0.01] *p* = 0.127	*ß* = 0.01 [−0.04; 0.06] *p* = 0.693	*ß* = 0.01 [−0.03; 0.05] *p* = 0.616	*ß* = −0.01 [−0.06; 0.03] *p* = 0.565	*ß* = 0.03 [−0.02; 0.08] *p* = 0.189	*ß* = −0.02 [−0.06; 0.02] *p* = 0.355

*Note:* Multivariate linear regression models adjusted for age and sex with standardized value of sleep spindles features (mean = 0 and standard‐deviation = 1). *ß* [95% CI] *p*‐value. Significant associations in bold.

^a^ For 10 unit.

## 4 Discussion

This was the first study to provide threshold values for central fast and frontal slow sleep spindle features—including duration, frequency, amplitude, density, frequential instability, and frequential purity—in healthy adults. These threshold measures can be utilized to identify pathology in diseased populations and to accelerate the discovery and diagnosis of neurological and psychiatric disorders. The significant differences between central fast and frontal slow spindles in all features underscore the importance of distinguishing between them.

As age advances, particularly from age 40 onwards, the duration, amplitude, and density of central fast and frontal slow sleep spindles decrease. Other features, such as frequential instability, also vary with age. Females exhibited higher amplitudes of both central fast and frontal slow spindles compared to males. After adjusting for age and sex, evening‐oriented participants showed longer central fast spindles, denser frontal slow spindles, and purer frequency of both spindle types. In addition, both types of spindle in participants with a higher proportion of slow‐wave sleep had higher amplitudes and their frontal slow spindles had lower frequencies. Our findings are in line with Adra et al. [46], who also reported specific differences between central fast and frontal slow spindles, including the shorter duration and higher density of central fast spindles compared to frontal slow spindles. However, they reported that fast spindles had lower amplitudes than slow ones, whereas we observed higher amplitudes for fast spindles. In addition, we noted greater frequential instability and greater deviation from the relative power of the sigma band (as measured via frequential purity) in central fast spindles. Our findings regarding age‐related differences in spindle features align with previous studies that have used various automated methods for sleep spindle detection and features analysis. These studies have consistently shown that spindle density, amplitude, and duration decrease from middle to late adulthood [2, 25, 47, 48, 49]. A reanalysis of three longitudinal studies, spanning from childhood to late adulthood, demonstrated reductions in spindle density (12%–15%), amplitude (5%–12%), and duration (2%) over a 5‐year follow‐up period [25]. Our results confirm that these age‐related changes affect both central fast and frontal slow sleep spindles.

Although some reports indicate that spindle frequency is minimally affected by aging [49] or remains relatively stable throughout adulthood and aging, our results show that, with age, the frequency of fast spindles increases while that of slow spindles remains unchanged, as partially observed by Djonlagic et al. [28]. For the first time, our study demonstrates that with age, the frequencies of both central fast and frontal slow spindles become increasingly unstable, and the frequency of frontal slow spindles moves closer to the relative power of the sigma band.

Previous studies have highlighted the significance of sleep spindles, particularly slow ones, for various sleep and wake functions, including memory consolidation and cognitive ability [2]. In addition, sleep spindles are altered in several neurological and psychiatric disorders [2]. For example, patients with chronic insomnia disorder exhibit higher spindle frequency and density [50] but shorter durations [51], whereas those with mild cognitive impairment or dementia show reduced amplitudes [52]. In line with these findings, we found that EDS was associated with increased amplitude in central fast spindles, despite all participants being free of sleep or neuropsychiatric disorders. We hypothesize that this relationship is not due to sleep loss, as experimental sleep deprivation is associated with decreased spindle amplitude in adolescents [53]. Conversely, insomnia has been linked to reduced spindle amplitude in previous research [41]. These correlations warrant further investigation to understand the origins of these sleep complaints and their potential links with spindle amplitude. Our study contributes values for various sleep spindle features, providing thresholds to differentiate between normal and pathological conditions. Future research is needed to evaluate the predictive value of these thresholds and their role in the management of these disorders, including initial evaluation and ongoing monitoring.

Circadian regulation of sleep spindles has been well‐documented, with studies showing that spindle density increases when sleep occurs at the optimal circadian time [54]. In our study, evening chronotypes exhibited lower density in frontal slow sleep spindles, which may be attributable to slight circadian misalignment imposed by laboratory constraints, consistent with previous research [55]. The absence of effect in fast sleep spindles may be due to the low level of delayed phase in this healthy population (only one participant with a social jetlag over 120 min). Our study extends these findings by demonstrating that not only spindle density but also duration and frequential purity vary across chronotypes, potentially impacting sleep and wake functions in different ways. Sleep spindles are also subject to homeostatic regulation [2]. Unlike N3 sleep, spindle density decreases following sleep deprivation [54] and increases after napping [56]. This homeostatic regulation primarily affects spindle density rather than other characteristics [57]. The proportion of N3 sleep was positively correlated with the density of frontal slow spindles but, surprisingly, not with that of central fast spindles. Indeed, previous studies suggest that fast spindles may be more influenced by circadian regulation, while slow spindles are more strongly affected by homeostatic regulation [58, 59]. In addition, the amplitudes of both types of spindles increased with the proportion of N3. This is particularly significant since N3 and sleep spindles have their own physiological mechanisms, but both naturally decrease with age and are known biomarkers of cognitive impairment [2, 60].

Our study had several strengths. First, we provide the first threshold values for sleep spindles in a healthy adult population free of sleep or neuropsychiatric disorders and psychotropic medications. This builds on recent work establishing normative values in children and adolescents [24], which reported a median density of almost 7 per min and a median duration of almost 1.5 s at age 18, whereas we found a median density of almost 6 per min and a median duration of almost 1 s at age 20. It is important to acknowledge that these values may vary depending on the EEG recording method (intracranial vs. scalp), the type of EEG leads used for detection (central vs. frontal) and the spindle detection approach (instantaneous frequency measurement vs. Fourier transformation). However, these threshold values offer the advantage of being derived from a non‐invasive, widely accessible, and easy‐to‐use method. Ultimately, they could serve as normative values for a given scalp EEG detection method if they demonstrate reliability (i.e., replication under different conditions and across various populations) and validity (i.e., association with specific neurological or psychiatric disorders). Second, central fast and frontal slow sleep spindles were analyzed separately, accounting for their distinct sources based on differences in genetic determinants, spatiotemporal appearance, pharmacological regulation, hemodynamic correlates, and roles in memory formation [2]. We identified significant differences in the features of central fast and frontal slow spindles and their associations with other sleep parameters. However, a reanalysis of a large cohort (> 11,000 individuals) suggested that a continuous spectrum of spindle frequencies might better characterize these phenomena [25]. Third, in addition to confirming previous findings regarding age‐ and sex‐related changes in sleep spindle features—such as decreased duration, amplitude, and density with age [25], and higher amplitude in females [61, 62]—we introduced the two new features of frequential instability and frequential purity. We found that spindle frequency was altered with age and confirmed the trends toward reduced duration, amplitude, and density [25]. Furthermore, we found that females had a higher spindle amplitude [61, 62], and that the frequency of central fast and frontal slow spindles became increasingly unstable with age. Fourth, by examining the associations between sleep spindles and various subjective and physiological sleep parameters, we have generated new hypotheses about their physiological functions and potential applications in sleep medicine. Further studies are needed to explore the impact of such variation on related functions such as memory consolidation and cognitive abilities.

However, this study also had several limitations. First, our sample size did not allow us to provide threshold values stratified by age and sex, which may be essential to better distinguish between normal and pathological conditions in various populations. Nevertheless, the variation related to age and sex is likely minimal compared to the anomalies observed in neurological or psychiatric disorders. For example, the median duration of spindles in patients with chronic insomnia disorder has been reported to be 0.60 s [51], significantly below the extreme thresholds identified in our study. Second, our study was based on the analysis of a single night of sleep. Although the features of fast spindles are generally consistent across nights, assessing the duration and density of slow spindles may require more than one night of data [63]. Third, exclusion criteria selected a homogenous and healthy population, which may have limited the statistical power of the association analyses between spindle features and physiological sleep parameters. Fourth, alcohol, tobacco, and caffeine consumption were not formally prohibited in our study despite their possible influence on sleep spindles [64, 65, 66]. Nevertheless, this choice was made to avoid over‐selecting our population or inducing withdrawal, which would also have had an effect on sleep spindles [67]. Moreover, substance dependence was an exclusion criterion and as a result, consumption rates were low (i.e., no tobacco use, a median coffee consumption of 1 cup per day, and a median alcohol consumption of 2 drinks per week). Finally, additional statistical analysis found no significant correlations (*p* > 0.05) between coffee and alcohol consumption and spindle features. Fifth, the cross‐sectional and observational nature of our analysis limits the interpretation of the directionality and causality of the associations we observed. Future studies that use experimental and longitudinal designs are needed to determine how extreme sleep spindle features might predict sleep changes and conversely, how inappropriate sleep behaviors or untreated sleep disorders could disrupt sleep spindles.

In conclusion, sleep spindles are among the most heritable sleep EEG signatures, exhibiting significant variability in their appearance. This study provides the first threshold values for sleep spindles in healthy adults and explores their determinants and associations with both subjective and physiological sleep parameters. Our findings underscore the importance of studying sleep spindle characteristics under both normal and pathological conditions to develop innovative biomarkers for neurological and psychiatric disorders and to deepen our understanding of the sleeping human brain.

## Author Contributions

Conceptualization: Julien Coelho, Jean‐Arthur Micoulaud‐Franchi, Pierre Philip, and Jacques Taillard. Data curation: Jacques Taillard, Patricia Sagaspe, and Emmanuel d'Incau. Formal analysis: Julien Coelho, Jacques Taillard, Heloïse Degros, and Christian Berthomier. Funding acquisition: Jacques Taillard and Pierre Philip. Investigation: Julien Coelho, Paul Galvez, Jean‐Arthur Micoulaud‐Franchi, and Pierre Philip. Methodology: Jacques Taillard and Pierre Philip. Project administration: Jacques Taillard. Resources: Jacques Taillard, Jean‐Arthur Micoulaud‐Franchi, and Pierre Philip. Software: Julien Coelho and Jean‐Arthur Micoulaud‐Franchi. Supervision: Jacques Taillard and Pierre Philip. Validation: Jacques Taillard and Pierre Philip. Visualization: Julien Coelho, Jean‐Arthur Micoulaud‐Franchi, and Jacques Taillard. Writing – original draft: Julien Coelho, Jacques Taillard, and Jean‐Arthur Micoulaud‐Franchi. Writing – review and editing: Jacques Taillard, Jean‐Arthur Micoulaud‐Franchi, Pierre Philip, Paul Galvez, Patricia Sagaspe, Heloïse Degros, Emmanuel d'Incau, Christian Berthomier, and Julien Coelho.

## Conflicts of Interest

The authors declare no conflicts of interest.

## Data Availability

The data that support the findings of this study are available on request from the corresponding author (JC). The data are not publicly available due to privacy or ethical restrictions.
